# A Candidate Therapeutic Monoclonal Antibody Inhibits Both HRSV and HMPV Replication in Mice

**DOI:** 10.3390/biomedicines10102516

**Published:** 2022-10-08

**Authors:** Hugues Fausther-Bovendo, Marie-Eve Hamelin, Julie Carbonneau, Marie-Christine Venable, Liva Checkmahomed, Pierre-Olivier Lavoie, Marie-Ève Ouellet, Guy Boivin, Marc-André D’Aoust, Gary P. Kobinger

**Affiliations:** 1 Department of Microbiology and Immunology, Faculty of Medicine, CHU de Quebec and Laval University, Quebec City, QC G1V 4G2, Canada; 2 Global Urgent and Advanced Research and Development, Batiscan City, QC G0X 1A0, Canada; 3 Medicago R&D Inc., Québec City, QC G1V 3V9, Canada; 4 Galveston National Laboratory, University of Texas Medical Branch, Galveston, TX 77555-0610, USA

**Keywords:** HMPV, HRSV, therapeutic antibodies

## Abstract

Human metapneumovirus (HMPV) and human respiratory virus (HRSV) are two leading causes of acute respiratory tract infection in young children. While there is no licensed drug against HMPV, the monoclonal antibody (mAb) Palivizumab is approved against HRSV for prophylaxis use only. Novel therapeutics against both viruses are therefore needed. Here, we describe the identification of human mAbs targeting these viruses by using flow cytometry-based cell sorting. One hundred and two antibodies were initially identified from flow cytometry-based cell sorting as binding to the fusion protein from HRSV, HMPV or both. Of those, 95 were successfully produced in plants, purified and characterized for binding activity by ELISA and neutralization assays as well as by inhibition of virus replication in mice. Twenty-two highly reactive mAbs targeting either HRSV or HMPV were isolated. Of these, three mAbs inhibited replication in vivo of a single virus while one mAb could reduce both HRSV and HMPV titers in the lung. Overall, this study identifies several human mAbs with virus-specific therapeutic potential and a unique mAb with inhibitory activities against both HRSV and HMPV.

## 1. Introduction

The human metapneumovirus (HMPV) and the human respiratory virus (HRSV) are two ubiquitous respiratory viruses belonging to the *Pneumoviridae* family. Both viruses circulate within the human population worldwide (reviewed in [1,2]). Seroprevalence studies in European countries, Japan and the USA have indicated that more than 90% of children aged between 5 and 10 were previously infected with HMPV [3,4,5,6]. Similarly, studies in Europe, Kenya and India have indicated that HRSV seropositivity was above 95% in children of the same age range [6,7,8].

The HRSV and HMPV infections lead to similar symptoms, ranging from fever and coughing to wheezing, hypoxia, pneumonia and even death, in rare cases [4,9]. The HRSV and HMPV infections are more severe in immunocompromised individuals, including the elderly and younger children. Indeed, premature children and children with pre-existing conditions such as chronic heart or lung disease are especially vulnerable to these viruses [1,2].

The health and economic burden related to these viruses is illustrated by the facts that HRSV and HMPV are estimated to account for 28–57 % and 3–6%, respectively, of hospitalizations for acute respiratory tract infections in young children in Canada, France and China [9,10,11]. Furthermore, studies from Finland, South Africa and the USA suggest that HRSV and HMPV account for 15–30% and 4–12% of consultations for young children with upper or lower respiratory tract infections [12,13,14,15,16].

To date, there is no approved vaccine against HRSV or HMPV. There is also no licensed therapeutic drug against HMPV or HRSV. Palivizumab, a humanized monoclonal antibody (mAb), is used prophylactically in high-risk children including premature infants and children with pre-existing comorbidities such as chronic lung disease, bronchopulmonary dysplasia and congenital heart disease [17]. However, the efficacy of Palivizumab for preventing hospitalization is partial and estimated at 50% [18]. New therapeutics are therefore being developed against HRSV. Among them, two therapeutic antibodies including Nirsevimab and MK-1654 are currently in clinical phases of development [19,20]. In contrast, existing therapeutics against HMPV are still in the pre-clinical phases of development [21]. It is worth noting that although aerosol administration of ribavirin, a nucleotide analog, could be effective against HRSV and HMPV, the high cost of ribavirin and its documented side effects have prevented its widespread use [22].

Due to the limited therapeutic options for HRSV and HMPV infections, the development of novel treatments is required. Both HRSV and HMPV encode for a fusion (F) and a glycoprotein (G) that are important for viral entry. The G protein is highly divergent among HRSV and HMPV subtypes (groups A and B), while the F protein is highly conserved [23,24,25]. As a result, the F protein of both viruses is the most attractive target for the generation of therapeutic mAbs against HRSV and HMPV.

This study documented the isolation, using flow cytometry-based cell sorting, of therapeutic mAbs against HRSV and HMPV from healthy human donors. The capacity of the plant-based expression platform from Medicago to effectively express the isolated mAbs was also assessed and the binding affinity and neutralization capacity of the resulting mAbs were evaluated in vitro by enzyme-linked immunosorbent assay (ELISA) and micro-neutralization, respectively. Finally, the ability of the selected mAbs to reduce lung viral loads was evaluated in murine models of HRSV and HMPV.

## 2. Materials and Methods

### 2.1. Human Samples

Blood samples from healthy human donors were obtained from the University Institute of Cardiology and Respirology of Quebec (IUCPQ). Signed informed consent was obtained from every study participant. Ethical approval (#2018-2982) for the conduct of this study was obtained from the ethics committee of the Université Laval-CHU de Quebec.

### 2.2. HMPV and HRSV Fusion Protein Production

The HMPV WT F, RSV WT F and RSV PreF genes were amplified and cloned in vector pcDNA 3.1+ (ThermoFisher Scientific, Burlington, ON, Canada, cat#V790-20). The HMPV and HRSV F proteins originated from the C-85473 and A2 strains, respectively. The RSV PreF protein was generated as previously described by introducing the S155C-S290C (DS) and S190F-V207L (CAV1) mutations (Figure S2) [26]. Plasmids were transfected in Expi293F cells using ExpiFectamine 293 reagent in order to express the different proteins, according to the manufacturer’s instructions. On day 5 post-transfection, supernatant containing the protein was recovered and proteins were then purified with Ni-NTA agarose (Qiagen, Toronto, ON, Canada, #30230).

### 2.3. Isolation of HMPV and RSV Specific B Cells

The HMPV and RSV fusion proteins were fluorescently labelled using the Alexa Fluor 488 and 647 antibody labelling kit (ThermoFisher Scientific), respectively, according to the manufacturer’s instructions.

The PBMCs were separated from healthy donors’ blood samples by Ficoll and frozen until needed. The PBMCs were thawed and incubated with 2 μg each of fluorescently labelled HMPV and RSV fusion protein. After 30 min incubation, samples were stained with a combination of lineage markers including antibodies against CD3 (SP34-2), CD19 (HIB19), IgM (G20-127), IgD (G18-145) all from BD Biosciences (San Jose, CA, USA). Dead cells were excluded using the fixable viability dye eFluor 780 (ThermoFisher Scientific).

The RSV- and HMPV-specific B cells were individually sorted using a BD FACS ARIA Fusion (BD Biosciences). The variable domain of heavy and light chains from the sorted B cells was amplified by RT-PCR as previously described [27]. Amplified fragments were sequenced using the Sanger techniques at the sequencing platform from the CHU de Quebec-Université Laval.

### 2.4. Monoclonal Antibody Production and Characterization

Cloning of heavy and light chains and expression in the *Nicotiana benthamiana* transient expression system was performed as previously described [28], with the following modifications. To maximize expression, 2X35S promoter linked to CPMV RNA-2 5′UTR coupled with CPMV RNA-2 3′UTR linked to NOS terminator in a vector containing the TBSV P19 suppressor of silencing under the control of Plastocyanin promoter and terminator was used. For infiltration, Agrobacterium suspensions containing vectors expressing the appropriate heavy and light chains were mixed at a final bacterial OD600 of 0.5. For IgG purification, no pH drop and TFF steps were performed and 1 mL of MabSelect SuRe LX™ (Cytiva) was added directly to clarified crude extract and agitated for 30 min. For in vivo study, neutralized mAb preparations were buffer-exchanged in PBS buffer using Amicon 100 kDa centrifugal devices (EMD Millipore) and sterilized using a 0.45/0.2 µg syringe filter (EMD Millipore). Total protein was analysed using Bradford Assay, purity was analysed using image densitometry on Coomassie-stained SDS-PAGE gel, endotoxin level was analysed using Endosafe^®^ nexgen-MCS™ (Charles Rivers) assay and finally, sterility was assessed by plating the mAb solution on TSA medium and incubation at 30 °C for 7 days.

### 2.5. HMPV ELISA

Wells were coated overnight at 4 °C with 60 ng of purified HMPV fusion protein. The next day, plates were washed and blocked with PBS, 5% milk. An hour later, wells were incubated with diluted antibodies for an additional hour. After extensive washes, samples were incubated with 1.5 ng of HRP conjugated anti human IgG. Absorbance was read at 405 after 30 min of incubation with ABTS substrate (Mandel scientific, Guelph, ON, Canada).

### 2.6. HRSV ELISA

Wells were coated overnight with 50 ng of goat anti-His antibodies (Novus Biological, Toronto, ON, Canada). Wells were extensively washed, then blocked for an hour with PBS, 5% milk. Wells were incubated at 37 °C with 250 ng of RSV fusion protein for 60 min, prior to another incubation with diluted antibody. After additional washes, wells were incubated with 1.5 ng of HRP-conjugated human cross-adsorbed with anti IgG (Thermofisher Scientific). Antibody binding was detected by measuring the absorbance at 405 after an incubation with ABTS substrate (Mandel scientific).

### 2.7. HRSV and HMPV Viruses

The HRSV and HMPV strains were grown on Hep-2 and LLC-MK2 cells, both from ATCC (Manassas, VA, USA) and concentrated by ultracentrifugation as previous described [29]. The HMPV C-85473 and RSV-A2 strains were used for in vivo challenge, while the HRSV strains 23094 and 22909, as well as the HMPV strains 16155 and 17480, were used for microneutralization.

### 2.8. HRSV and HMPV Microneutralization

Serial dilutions of the different mAbs starting at 10 µg/mL were performed in order to determine their capacities for neutralizing HMPV and HRSV strains. Sixty-five PFU of HMPV-A (16155), -B (17480), RSV-A (23094) or -B (22909) viruses were added to each dilution of mAb for a co-incubation of 1 h at 37 °C. After the co-incubation period, the mixtures were transferred on LLC-MK2 cells to allow an infection for 1 h 30 at 37 °C. Following the infection, the mixtures were removed and replaced with fresh media. Viral titers were revealed by immunostaining after a 3 day incubation period at 37 °C. Palivizumab was used as a positive control for microneutralization against RSV.

### 2.9. In Vivo Efficacy

Four to six-week old female Balb/c mice were purchased from Charles River (Quebec, Canada). For each group, 10 mice were challenged intranasally with 5 × 10^5^ TCID_50_ of HMPV (C-85473 strains) and 1 × 10^7^ PFU of HRSV (A2 strain). Twenty-four hours post- challenge, mice were either mock-treated or received 250 μg (15 mg/kg) of antibody. Four mice per group were euthanized on days 4 and 5 to measure lung viral titers. Animals were individually weight for 14 days post viral challenge.

### 2.10. Lung Viral Titers

Lung viral titers were determined by plaque assay on day 4 and 5 post-infection for HRSV and HMPV, respectively. Lungs were removed and homogenized in 1 mL of PBS. The homogenates were serially diluted 1/10 in media and lay down on LLC-MK2 cells for an incubation of 1 h 30 at 37 °C. After the incubation period, the homogenates were removed and replaced by fresh media containing 0.8% methycellulose. Lung viral titers were determined by immunostaining after an incubation of 3 days at 37 °C.

### 2.11. Statistics

Kruskal–Wallis test followed by the Dunn’s test was used to lung viral titer better mock and mAb-treated mice. ** and * represent *p* values < 0.01 and 0.05, respectively. All statistical analyses were performed using GraphPad Prism software.

## 3. Results

### 3.1. Isolation of HRSV and HMPV Fusion Protein Specific B Cells

**Figure 1:**
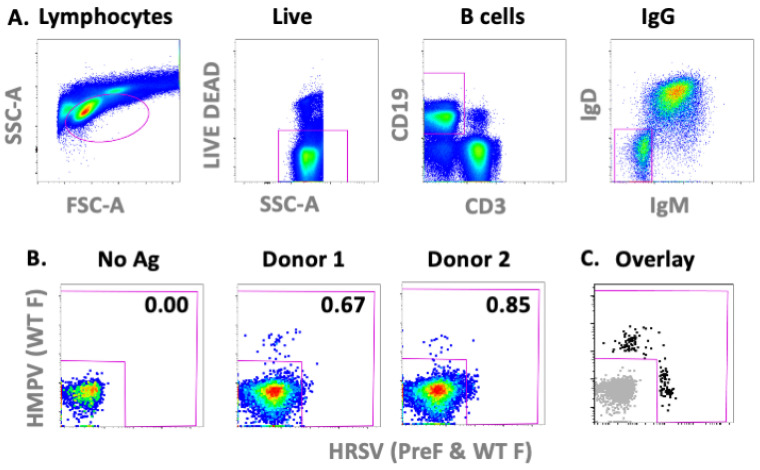
Isolation of HMPV and RSV fusion-specific B cells. Virus-specific B cells were isolated using flow cytometry-based cell sorting. The PBMCs from healthy donors were stained using fluorescently labeled HMPV and RSV fusion protein as well as lineage markers. (**A**) The gating strategy for B cells isolation is illustrated. (**B**,**C**) Representative plots from the IgG-positive B cells (Live/Dead low, CD19+ CD3− IgD− IgM−) gate are depicted. (**B**) Unlabelled samples (left) and stained healthy donors (right) are shown. (**C**) Representative sorted B cells (black) were overlaid onto unlabelled B cells (grey).

This study aimed to develop new therapeutic mAbs against HMPV and HRSV. Both viruses are ubiquitous, with every individual encountering these viruses during childhood. As a result, healthy human donors were used to isolate monoclonal antibodies. Peripheral Blood Mononuclear Cells (PMBCs) were isolated from 14 healthy donors under appropriate regulatory, clinical and ethical oversight from Université Laval-CHU de Quebec. The B cells specific to HRSV and HMPV Fusion (F) proteins were isolated using flow cytometry-based cell sorting. To this end, wild-type F proteins (WT F) from both viruses and a pre-fusion F protein from HRSV (PreF) were fluorescently labelled. The fluorescent proteins were combined with lineage markers for live IgG-positive B cells to identify antigen-specific B-cells within PBMC samples (Figure 1). These individually sorted B-cells underwent nested PCR to amplify the variable region of their heavy and light chains. Matching light and heavy chain sequences were obtained for 102 mAbs.

### 3.2. Production and Characterization of Binding Affinity against HMPV and HRSV

**Figure 2:**
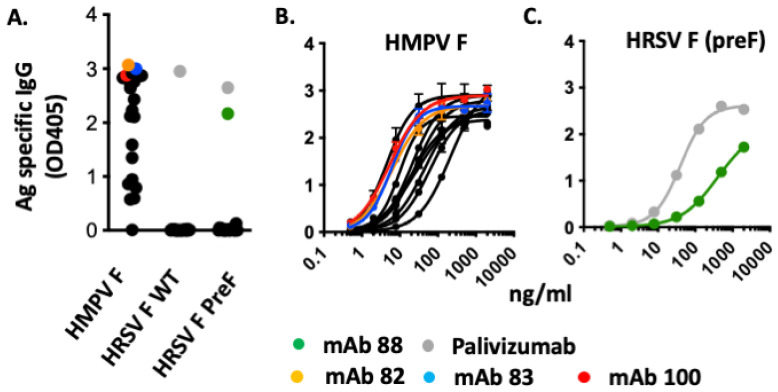
Binding efficiency of HMPV and RSV-specific mAbs. The binding efficiency of purified mAbs was analysed by ELISA. (**A**) MAb binding efficiency against HMPV and RSV fusion proteins was first analysed at 2 μg/mL antibody. For RSV, binding to the wild-type (WT) and Pre Fusion (PreF) form of the fusion protein was used. MAb (*n* = 21) with sufficient binding (OD above 0.5) against HMPV or RSV are depicted in black, while Palivizumab is illustrated in grey. (**B**,**C**) Binding affinity against HMPV and RSV fusion proteins was analysed using serial dilutions of selected mAbs (*n* = 12 for HMPV and *n* = 1 for RSV preF). mAb 82 (yellow), mAb 83 (blue), mAb 88 (green) and mAb 100 (red) are highlighted. Palivizumab (grey) was used as positive control against RSV.

Each of the 102 matching light and heavy chains were transiently co-expressed in *Nicotiana benthamiana* by agroinfiltration and screened for expression using Sodium Dodecyl Sulphate-Polyacrylamide Gel Electrophoresis (SDS-PAGE) analysis of clarified protein extracts. Of these, 95 mAbs were successfully expressed and purified by protein A and the binding affinity of the 95 produced mAbs was evaluated by ELISA. First, each antibody was tested at a single concentration (2 μg/mL) using plates coated with HMPV WT F, HRSV WT F as well as HRSV PreF protein. Of the 95 produced mAbs, 21 showed significant binding to HMPV WT F, while a single mAb was found to bind to HRSV PreF but not to HRSV WT F. In contrast, Palivizumab strongly bound to both HRSV WT F and HRSV PreF (Figure 2A).

Next, based on the highest optical density (OD), the binding strength of the top 12 mAb candidates was determined by ELISA using serial dilutions. The half maximal effective concentration (EC_50_) of the top 4 mAb candidates (namely, mAb 29, 82, 83 and 100) against HMPV WT F ranged from 4.4 to 6.5 ng/mL, while the EC_50_ of the remaining antibodies was between 9.3 and 214.8 ng/mL (Figure 2B). Against HRSV PreF, the only mAb with significant binding (namely, mAb 88) had an EC_50_ of 414 ng/mL. In contrast, Palivizumab EC_50_ was around 38.8 ng/mL against the same target (Figure 2C).

### 3.3. Neutralization Capacity of Antibodies with Strong Binding Affinity

In vitro neutralization tends to be associated with in vivo protective efficacy [30]. Therefore, the neutralization capacity of the 12 mAbs with the highest binding efficiency against HMPV WT F and mAb 88, the only mAb that recognized HRSV PreF, was measured at 10, 1 and 0.1 μg/mL using a microneutralization assay. Neutralization against clinical isolates representing the two groups A and B of both HMPV and HRSV was performed for the above mAbs. Palivizumab was used as a positive control for HRSV.

Of the 13 mAbs tested, 4 mAbs (mAb 101, 94, 95, 99) did not neutralize any of the 4 tested viruses, while 3 mAbs (mAb 88, 89, 90) displayed some neutralization against HRSV. This finding was surprising as two of these mAbs (89 and 90) did not show any significant binding against RSV Fusion proteins (WT or PreF) by ELISA. The final mAb (mAb 88) demonstrated strong neutralisation against both HRSV A and B with half maximal inhibitory concentrations (IC_50_) of 13.2 and 30.6 ng/mL, respectively. In contrast, Palivizumab IC_50_ was 216.5 and 129.5 ng/mL against the same HRSV virus (Table 1).

Eight mAbs were able to neutralize the HMPV isolates tested. Of these mAbs, 82 and 83 showed the strongest neutralization capacity against HMPV A and HMPV B. Additional mAb dilutions were needed to determine their IC50. mAb 82 IC_50_ against HMPV A and B was 13.9 and 1.7 ng/mL, respectively, while mAb 83 IC_50_ against the same viruses was 17.2 and 9.5 ng/mL (Table 1).

### 3.4. In Vivo Inhibition of HRSV and HMPV

Based on the ELISA and neutralization data, the inhibitory activity of the best mAb candidates against HMPV and HRSV was further evaluated in vivo. MAbs 82, 83 and 88 were selected due to their strong neutralization activity against HMPV or HRSV, while mAb 100 was chosen for its strong binding affinity against HMPV WT F. The four mAbs were purified by protein A and, after buffer exchange and sterilization filtration, mAbs were characterized for protein content, product quality, bioburden and endotoxin level. All purified mAbs showed high protein content (≥1 mg/mL), low residual endotoxin levels (≤0.4 EU/µg protein), sterility (<10 Colony Forming Units/mL), high purity (≥98%) and met all release criteria for mouse administration. Mice were infected with either 10^7^ PFU of HRSV or 5 × 10^5^ 50% Tissue Culture Infectious Dose (TCID_50_) of HMPV using previously established conditions specific to each murine model [31,32]. Twenty-four hours after challenge, mice were treated intraperitoneally with 250 μg of each selected mAb of interest. The PBS- or Palivizumab-treated mice were used as negative or positive control, respectively. On day 4 post-HRSV or day 5 post-HMPV infection, 4 out of 10 mice of each group were culled to measure lung viral titers. The weight loss of the remaining mice was monitored for 14 days post challenge.

During the first days post-HMPV challenge, mock-treated animals lost between 2 and 6% of their weight and animals quickly recovered afterwards. No significant difference in weight loss was observed between the mock- and antibody- treated animals at the various time points (Figure S1). Similar results were observed in HRSV-challenged animals, with weight loss averaging 11.5 and 4.5% on days 1 and 2 post-challenge in mocked-treated, and animals gaining weight thereafter. Once again, no statistically significant difference in weight loss was observed between mock-treated and antibody- treated animals (Figure S1).

**Figure 3:**
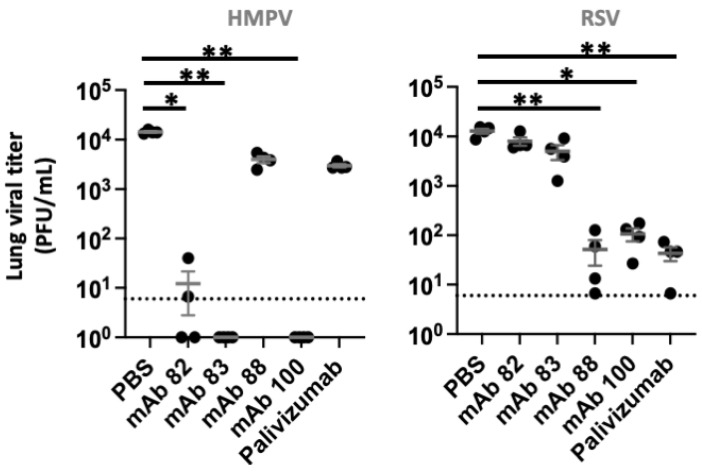
In vivo efficacy of selected mAbs against HMPV and HRSV. Female Mice were challenged with 5 × 10^5^ TCID_50_ of HMPV or 1 × 10^7^ PFU of HRSV. Twenty four hours post-infection, mice received PBS or 250 μg of mAb. On days 4 and 5 post- HRSV and HMPV infections, respectively, viral lung titers were assessed (*n* = 4/group). *, ** indicates a statistically significant difference (*p* < 0.05, <0.01). Mean +/− SEM are shown.

Lung viral titers, which provide a more accurate measure of the therapeutic efficacy of mAbs, were then analyzed. Treatment of HMPV-infected mice with Palivizumab or mAb88 resulted in modest decreases in lung viral titers. In contrast, treatment of HMPV-infected animals with Mabs 82, 83 or 100 resulted in an over 1000-fold decrease to below the limit of detection of the assay in lung viral titers (*p* values below 0.05, 0.01 and 0.01, respectively; Figure 3). In HRSV-infected mice, treatment with mAb 82 or 83 only resulted in negligible reduction in lung viral titers. In contrast, treatment with mAbs 88, 100 or Palivizumab led to a more than 100-fold reduction in lung viral titers in all treated animals relative to controls (*p* values < 0.05, < 0.01, < 0.01, respectively; Figure 3).

## 4. Discussion

The HRSV and HMPV viruses cause a substantial disease burden. Each year, both viruses account for millions of infections and hospitalizations worldwide [9,10,11]. Children younger than 5 years old are especially susceptible to these infections. As a result, the development of novel prophylactic and therapeutic options is required to alleviate the burden caused by these viruses.

In this study, we performed the isolation, production and evaluation of one hundred human monoclonal antibodies against HMPV, HRSV, or both. Most of the mAbs isolated were against HMPV, suggesting that the current isolation protocol favored this target and that modifications would be required to prioritize the identification of mAbs against HRSV. It is worth pointing out that, based on our ELISA results, only a single mAb (mAb 88) was isolated against HRSV. However, two additional mAbs (89 and 90) were able to neutralize HRSV in vitro while mAb 100 reduced HRSV lung titer in vivo. This indicates that the in-house ELISA assay used in this study was unable to detect several HRSV-specific mAbs. Of note, the F proteins of both HMPV and HRSV form trimers on viral particles and on the surface of infected cells [33,34]. Flow cytometry may allow the isolation of mAbs targeting more conformational epitopes involving more than monomer. These complex epitopes would be present on viral particles but rare in serological assays. Additional studies on the binding epitopes of the isolated HRSV-specific mAb would be required to better understand their profiles and inform on the need to include more complex conformational epitopes during mAb screening.

The present study confirmed that protective mAbs against both viruses exist and can be isolated from healthy donors. One of our isolated antibodies, mAb 100, was able to reduce the lung viral titers of mice challenged with either HRSV or HMPV. To our knowledge, this is the third human mAb that has been shown to prevent the in vivo replication of both viruses. Previously, the human mAbs MPE8 and 54G10 were shown to reduce viral lung titers in mouse models of HRSV and HMPV infections [35,36]. Of note, additional mAbs cross-reactive to HRSV and HMPV have been reported recently. However, the protective efficacy of these mAbs is unknown [37,38]. MPE8 and 54G10 were reported to neutralize both HRSV and HMPV in vitro [35,36]. Although mAb 100 neutralized HMPV representatives of the two clades, no neutralization against any of the tested HRSV viruses was noted. Interestingly, the ability of mAb 100 to reduce HRSV viral load in vivo, despite the lack of in vitro neutralization, highlights the importance of other antibody-mediated function(s) in the control of virus propagation. In vivo, non-neutralizing mAbs can limit viral replication through both Fab- and Fc-mediated mechanisms. First, non-neutralizing mAbs can act synergically with their neutralizing counterparts. Indeed, non-neutralizing mAbs were shown to increase the neutralization capacity of mAbs against the severe acute respiratory syndrome coronavirus 2 (SARS-CoV-2), Ebolavirus, Marburg virus and Mayaro virus, notably by improving access to neutralizing epitopes [39,40,41,42]. Second, non-neutralizing mAbs can also interact with natural killer (NK) cells and monocytes to promote viral clearance via antibody-dependent cellular phagocytosis (ADCP), antibody-dependent virus opsonization, antibody-dependent cellular cytotoxicity (ADCC) as well as antibody-induced monocyte maturation [43,44]. Recently, a predominant role of monocytes in the protection mediated by non-neutralizing mAbs was suggested based on results from animal models of SARS-CoV-2, Marburg and Mayaro virus infections [39,42,45].

In our challenge model of infections, mice were euthanized 4–5 days post-HRSV or HMPV challenge. As a result, non-neutralizing mAb cooperation was unlikely to play a significant protective role, suggesting a major contribution of Fc-mediated functions such as ADCP, ADCC, antibody-dependent virus opsonization and antibody-induced monocyte maturation. Understanding their relative contribution to mAb-mediated HRSV viral suppression will be important in the future. This study further supports the inclusion of non-neutralizing mAbs in pre-clinical screening of candidate mAbs. Indeed, protection of non-neutralizing mAbs was demonstrated in rodent models of H7N9 influenza, Ebola, Marburg, HCMV and Mayaro virus [30,39,42,46,47,48].

In this study, mAbs were administrated intraperitoneally to achieve systemic delivery. Although therapeutic antibodies are usually given intravenously, alternative routes of administration are currently being evaluated [49,50]. For respiratory infections, several aerosol delivery devices and chemical formulations have been developed for the delivery of therapeutic agents in the respiratory tract [49,51,52]. The novel mAb candidates identified here should also be evaluated within airway-delivery protocols in different animal species to better characterize their therapeutic potential. In summary, we have reported the isolation of several mAb-based therapeutic candidates against HMPV and HRSV as well as one mAb capable of significantly reducing both HRSV and HMPV viral replications in vivo. Our study warrants further investigation of the therapeutic potential of some of these mAb candidates in larger animal models and using various delivery routes.

## Figures and Tables

**Table 1:** In vitro neutralization of HMPV and HRSV strains by monoclonal antibodies. The neutralization capacity of selected monoclonal antibodies against HMPV and RSV was evaluated using microneutralization assays. Each antibody was tested at 10, 1 and 0.1 μg/mL against two separate strains of HMPV as well as two strains of RSV. -, +, ++, +++, ++++ indicate 0, 25, 50, 75 and 100% plaque reduction, respectively. Palivizumab [PZB(+)] was used as positive control against RSV.

mAbs	HMPV A-16155 (µg/mL)			HMPV B-17480 (µg/mL)			RSV A-23094 (µg/mL)			RSV B-22909 (µg/mL)		
	10	1	0,1	10	1	0,1	10	1	0,1	10	1	0,1
29	+++	+	-	++	++	-	-	-	-	-	-	-
82	+++	++++	+++	++++	++++	+++	-	-	-	-	-	-
83	++++	+++	+++	++++	+++	++	-	-	-	-	-	-
87	+++	+++	-	+++	+	-	-	-	-	-	-	-
88	-	-	-	-	-	-	++++	+++	++	+++	+++	++
89	+++	++	-	+	-	-	++	-	-	-	-	-
90	+++	++	++	+++	+	+	++	+	+	++	++	-
92	+++	++	-	+++	+	-	-	-	-	-	-	-
100	+++	++	-	+++	+	+	-	-	-	-	-	-
PZB(+)	-	-	-	-	-	-	+++	++	-	++++	+++	-

## Data Availability

The data presented in this study are available on request from the corresponding author.

## Supplementary Materials

The following are available online at https://www.mdpi.com/article/10.3390/biomedicines10102516/s1, Figure S1: Weight loss following HRSV and HMPV challenge, Figure S2: Schematic representation of HRSV and HMPV F proteins.
