# Supplementary material for: A Candidate Therapeutic Monoclonal Antibody Inhibits Both HRSV and HMPV Replication in Mice
Source: Biomedicines. 2022 Oct 8;10(10):2516. doi: 10.3390/biomedicines10102516 (PMC9599547; doi:10.3390/biomedicines10102516)
Supplement: Supplementary file 1 [file biomedicines-10-02516-s001.zip › biomedicines-1939172-supplementary.pdf]

Supplementary Figures

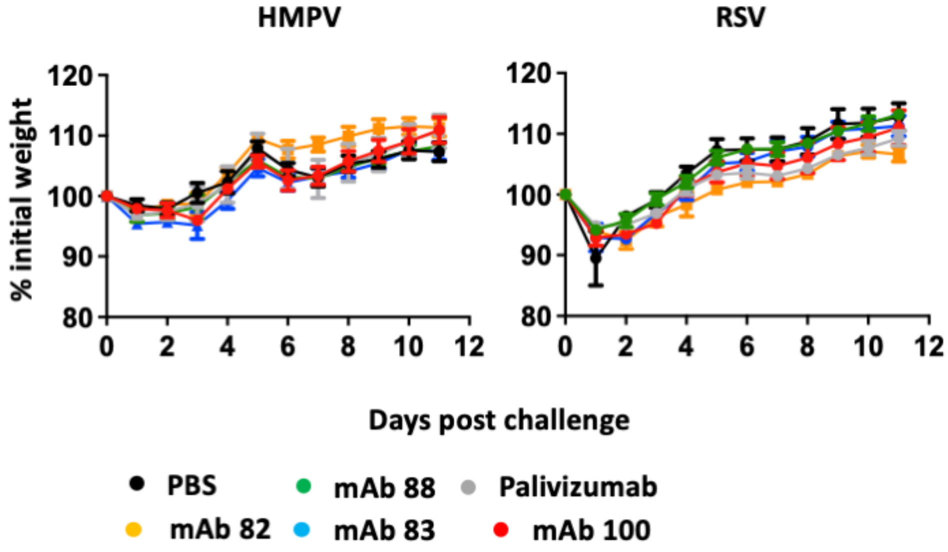

Figures S1: Weight loss following HRSV and HMPV challenge. Female Mice were challenged with $5x10^5$ $TCID_{50}$ of HMPV or $1x10^7$ PFU of HRSV. One day later, mice received PBS or 250 mg of mAb. Weight loss (n = 6/group) is depicted. No statistically significant difference (p >0.05) were observed between the groups. Mean+/-SEM are shown.

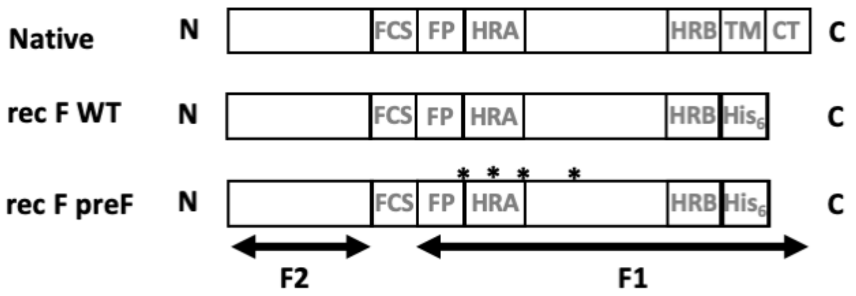

Figures S2: Schematic representation of HRSV and HMPV F proteins. The conserved features of the HRSV and HMPV F precursors in the N to C terminal orientation are depicted. Native proteins as well as the recombinant version (rec) including pre-fusion (pre-F) and wild-type (WT) used are illustrated. The F1 and F2 subunits are shown as well as the heptad-repeat (HR) A and B; the furin cleavage site (FCS), the fusion peptide (FP), the transmembrane domain (TM) and cytoplasmic tails (CT). Both the TM and and CT were deleted from the recombinant F proteins, while the S155C-S290C (DS) and S190F-V207L (CAV1) mutations indicated by asterisks (*) were introduced to obtained the HRSV pre-F proteins.
